# AI‐Based Prediction of PROTAC‐ and Molecular Glue‐Mediated Ternary Complexes: A Comparative Evaluation of AlphaFold 3 and Boltz‐2

**DOI:** 10.1002/ardp.70225

**Published:** 2026-03-14

**Authors:** Lino Riepenhausen, Anne‐Christin Sarnow, Dina Robaa, Wolfgang Sippl

**Affiliations:** ^1^ Department Medicinal Chemistry, Institute of Pharmacy Martin‐Luther‐Universität Halle‐Wittenberg Halle Saale Germany

## Abstract

Proteolysis targeting chimeras (PROTACs) and molecular glues induce ligand‐mediated ternary complexes between an E3 ubiquitin ligase and a protein of interest, but their in silico modeling remains challenging due to conformational flexibility and weak protein‐protein interfaces. Recent diffusion‐based AI structure prediction models enable the direct prediction of protein‐ligand complexes. Here we benchmarked AlphaFold 3 and Boltz‐2 for predicting PROTAC‐ and molecular glue‐mediated ternary complexes using a reproducible evaluation workflow. We curated a dataset of 40 experimentally resolved complexes from the Protein Data Bank, including 25 PROTAC and 15 molecular glue systems. Structural accuracy was assessed using complex RMSD and DockQ scores relative to the corresponding crystal structures and compared to model‐internal confidence metrics. Both models outperform other current approaches in both accuracy and runtime. Boltz‐2 shows higher prediction accuracy assessed by complex RMSD and DockQ scores. Predictions are generally more accurate for VHL‐based PROTACs than for CRBN‐based PROTACs. Predictions for molecular glue complexes show good overall accuracy. Error analysis indicates that prediction failures predominantly arise from misoriented global arrangements and twisting in flexible ternary complexes, while individual protein and ligand structures are often accurately modeled. Limitations in the generalizability of the models could also be observed, especially for more recently released structures. These findings suggest that diffusion‐based AlphaFold‐type models show promise in the structure‐based prediction of PROTAC‐ and molecular glue‐mediated ternary complexes.

## Graphical abstract

Superposition of the lowest RMSD predictions for the ternary complex of SMARCA2‐VHL. The AlphaFold 3 prediction is shown in cyan, the Boltz‐2 prediction in pink, and the reference X‐ray structure in green.

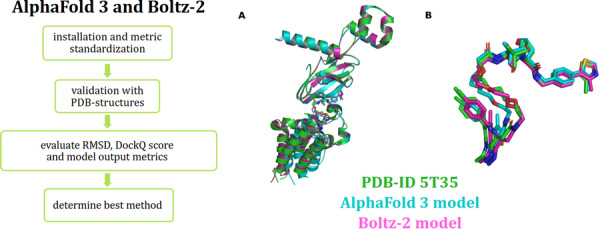

## 1 Introduction

Proteolysis targeting chimeras (PROTACs) are heterobifunctional molecules composed of an E3 ligase‐binding ligand, a protein of interest (POI)‐binding ligand and a flexible linker connecting them. Simultaneous binding of both proteins positions the E3 ligase in proximity to the POI, thereby facilitating ubiquitination and subsequent proteasomal degradation. Although no PROTAC has yet been clinically approved, several PROTACs, primarily targeting cancers, are in advanced clinical trials, with extensive efforts to expand their application to additional diseases [1]. They are large and conformationally flexible molecules with many rotatable bonds [2, 3]. In contrast to conventional inhibitors, they do not just occupy a single defined pocket, but rather induce a transient protein‐protein interface. Consequently, the ternary complexes have conformational flexibility, cooperative effects and entropic penalties, which make traditional docking methods unsuitable for modeling these systems [2, 4, 5].

Several computational PROTAC‐mediated ternary complex modeling methods have been published which include Molecular Operating Environment (MOE) based methods, ICM, PRosettaC and HADDOCK protocols among others [2, 4, 5, 6, 7]. Most of these approaches combine protein‐protein docking, degrader conformational sampling and clustering to identify geometrically compatible poses and require prior knowledge of the binary binding modes of both the E3 ligase and POI [2, 4]. Although these methods work successfully for some ternary complexes, their performance is inconsistent across systems and even the highest‐ranked complexes in clustering predictions often have root‐mean‐square deviation (RMSD) values greater than 4 Å [4]. They are also computationally expensive with reported runtimes ranging from tens to hundreds of CPU hours. For example, one study showed that PRosettaC requires about on average 369 CPU hours, MOE method 4B 53 h and ICM 15 h per PROTAC ternary complex [4]. In this work, we focus on von Hippel‐Lindau (VHL) and cereblon (CRBN) based PROTAC‐mediated ternary complexes, as these are currently the best studied of these systems [8].

Molecular glues are smaller monovalent ligands that stabilize otherwise weak or transient protein‐protein interactions. Several clinically used drugs that treat cancer, autoimmune disorders and infectious diseases were found to act as molecular glues [9]. Unlike PROTACs, they do not have a linker and instead often exert their effect allosterically. They can induce targeted protein degradation (TPD) by binding to an E3 ligase and creating a novel protein‐protein interface with a POI. This brings the POI into proximity with the E3 ligase, resulting in its ubiquitination and subsequent proteasomal degradation [9]. Despite their extensive medical relevance, historically their discovery has mostly been serendipitous [9].

Modeling these complexes is difficult because of their dynamic and poorly conserved induced interfaces across systems. Previously published approaches like YDS‐GlueFold and FKSFold were built on top of AlphaFold‐type architectures using techniques like guided diffusion or Feynman‐Kac‐guided sampling [9, 10, 11]. However, their predictions still have limited accuracy. Another problem is the scarcity of molecular glue‐mediated ternary complexes in the PDB, which limits both training data and benchmarking potential. The focus of this work is on CRBN‐based molecular glue‐mediated ternary complexes, as they are currently the best studied ones for molecular glues [9].

The objective of this work is to benchmark the performance of the AI‐based structure prediction models AlphaFold 3 and Boltz‐2 for modeling PROTAC‐ and molecular glue‐mediated ternary complexes (Table 1). AlphaFold 3 and Boltz‐2 belong to the class of diffusion‐based transformer models that can, in addition to predicting protein structure, also incorporate ligands and further biomolecules [12, 13]. They do not require predefined binary binding modes or manual cluster filtering, allowing direct prediction of complete ternary complexes, simplifying the modeling workflow.

**Table 1:** Comparison of AlphaFold 3 and Boltz‐2 based on core architecture, training data, configurability, and licence [12, 13].

Aspect	AlphaFold 3	Boltz‐2
Core architecture	48‐layer transformer backbone based on the AlphaFold 2 Evoformer, redesigned as a Pairformer with triangular attention on residue and atom pair representations, reduced MSA attention, frame‐agnostic diffusion module and recycling	64‐layer transformer backbone adapted from AlphaFold 3 Pairformer, SE(3)‐equivariant diffusion module, customizable recycling and optional physics‐based steering potentials and binding affinity prediction
Training data	PDB structures (Sep 30, 2021 cutoff) with cross‐distillation from AF‐Multimer 2.3	PDB structures (Jun 01, 2023 cutoff) with distillation from AFDB, Boltz‐1 and MD ensembles, additional binding‐affinity data
License	CC‐BY‐NC‐SA 4.0 (academic only, inference code only)	MIT License (fully open‐source)

## 2 Results and Discussion

### 2.1 Data set Selection

We curated a data set for benchmarking of the structure prediction capabilities of AlphaFold 3 and Boltz‐2 on TPD ternary complexes from the Protein Data Bank (PDB) up to September 2025 [14]. The complexes were selected to reflect the current experimental distribution of E3 ligases and POIs used in TPD. E3 ligases such as cIAP1 and BIRC2 for PROTAC‐mediated ternary complexes, and VHL for molecular glue‐mediated ternary complexes, were excluded due to insufficient structural coverage in the PDB to enable meaningful benchmarking. Additional selection criteria were a resolution of less than 4.0 Å and the exclusion of structures with incompletely resolved ligands. The final dataset comprised 40 ternary ligand‐mediated complexes. All of the selected complexes are shown in Table 2.

**Table 2:** Data set composition of PROTAC‐ and molecular glue‐mediated complexes across E3 ligases and proteins of interest (POIs) in the benchmarking data set, showing all ligase‐POI combinations.

PROTAC ternary complexes (25 total)									
E3 ligase	BRD4^BD1^	BRD4^BD2^	PTPN2	CDK2	SMARCA4	SMARCA2	FAK	WDR5	Total
CRBN	2	1	1	1	0	0	0	0	5
VHL	3	4	0	0	1	4	1	7	20

**Molecular glue complexes (15 total)**									
**E3 ligase**	IKZF1	IKZF2	GSPT1	WIZ	MBP‐SD40	SALL4	CSNK1A1		Total
CRBN	3	3	1	3	3	1	1		15

Abbreviations: CRBN, cereblon; PROTAC, proteolysis targeting chimeras; VHL, von Hippel‐Lindau.

The 25 PROTAC‐mediated ternary complexes, included five CRBN‐based and twenty VHL‐based systems covering diverse POIs. In addition, 15 CRBN‐based molecular glue‐mediated ternary complexes were included (Table 2). These entries represent nearly all CRBN molecular glue‐mediated ternary complexes deposited in the PDB up to October 2025 that met the defined selection criteria.

### 2.2 Structural Accuracy

Predicting five samples of PROTAC ternary complexes required approximately 15–25 min using AlphaFold 3 and 5–10 min using Boltz‐2. This difference is mainly due to differences in MSA computation: AlphaFold 3 relies on local database searches, whereas Boltz‐2 uses a remote MSA server.

**Figure 1:**
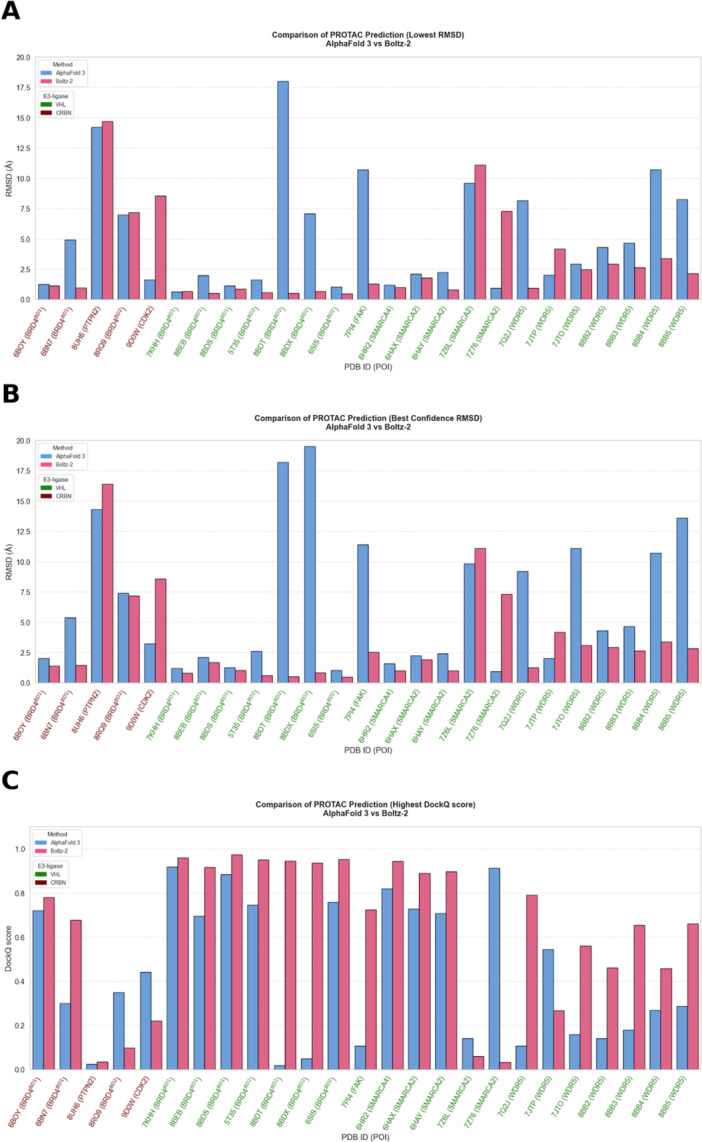
Comparison of AlphaFold 3 and Boltz‐2 performance on PROTAC‐mediated ternary complex predictions. Complex Cα‐RMSD is calculated relative to the experimental reference structure. (A) Minimum RMSD across all diffusion samples. (B) RMSD of the top‐ranked prediction according to the internal model confidence score. (C) Maximum DockQ score across all diffusion samples for each complex, evaluated against the experimental reference structure. PROTAC, proteolysis targeting chimeras; RMSD, root‐mean‐square deviation.

**Figure 2:**
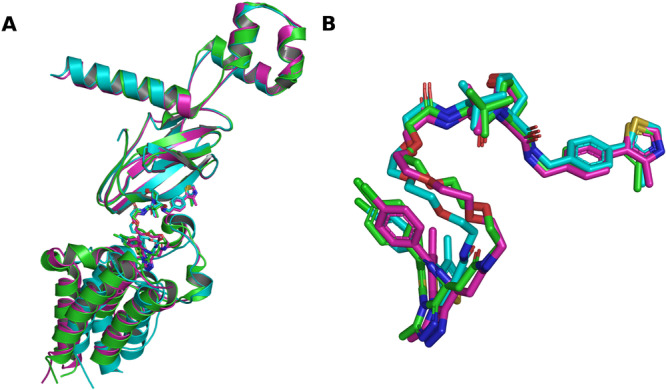
Superposition of ternary complex predictions for PDB ID 5T35. (A) complex superposition relative to the experimental reference structure, with proteins shown as cartoons and the PROTAC ligand as sticks. The POI (BRD4^BD2^) is shown at the bottom and the E3 ligase (VHL) at the top. (B) superposition focused on PROTAC ligands, shown as sticks. The reference crystal structure is shown in green, the AlphaFold 3 prediction in cyan, and the Boltz‐2 prediction in pink. The corresponding complex RMSD values are 1.62 Å for AlphaFold 3 and 0.58 Å for Boltz‐2. POI, proteins of interest; PROTAC, proteolysis targeting chimeras; RMSD, root‐mean‐square deviation; VHL, von Hippel‐Lindau.

The accuracy of the predicted models was assessed by calculating the Cα‐RMSD relative to the experimental reference structure after superposition. In addition, interface quality was evaluated using DockQ v2 to provide an interface‐sensitive assessment complementary to global RMSD (Figure 1). Both models achieved reasonably high predictive accuracy (e.g., observed for complex 5T35 shown in Figure 2), with Boltz‐2 producing overall slightly lower complex RMSD values. DockQ score trends were largely consistent with the RMSD‐based evaluation, confirming that improved global agreement generally coincided with more accurate interfacial geometry. However, some discrepancies were observed. For example, in the AlphaFold 3 predictions for 8BB2 and 8BB3, moderate RMSD values were obtained, whereas the corresponding DockQ scores were comparatively low. This discrepancy reflects the stronger emphasis of DockQ on interface accuracy, as opposed to the rigid‐body deviations primarily captured by RMSD. We observed larger performance differences for the complexes 6BN7, 8BDT, 8BDX, 7PI4, 7Q2J, 8BB4, and 8BB5, where AlphaFold 3 underperformed compared to Boltz‐2, and for 9D0W and 7Z76, where Boltz‐2 underperformed relative to AlphaFold 3. For most of the other complexes, both models show comparable results.

When the top‐ranked predictions according to the internal confidence score were evaluated by RMSD, Boltz‐2 still performs better overall and shows higher consistency between internal ranking and structural accuracy. AlphaFold 3 exhibits larger deviations between confidence‐based ranking and structural quality, particularly for 8BDX, 7JTO, and 8BB5. We observed that VHL‐based complexes are generally more accurately predicted than CRBN‐based ones, an effect that is further discussed in the limitations section.

We compared the lowest RMSD predictions from AlphaFold 3 and Boltz‐2 with the structures reported to have the lowest prediction RMSD for HADDOCK, ICM, MOE method 4B and PRosettaC in a previous benchmark study [4] (Supporting Information: Figure 2). Both AlphaFold 3 and Boltz‐2 achieve significantly lower RMSD values than all other evaluated methods and the inference times are also heavily reduced.

**Figure 3:**
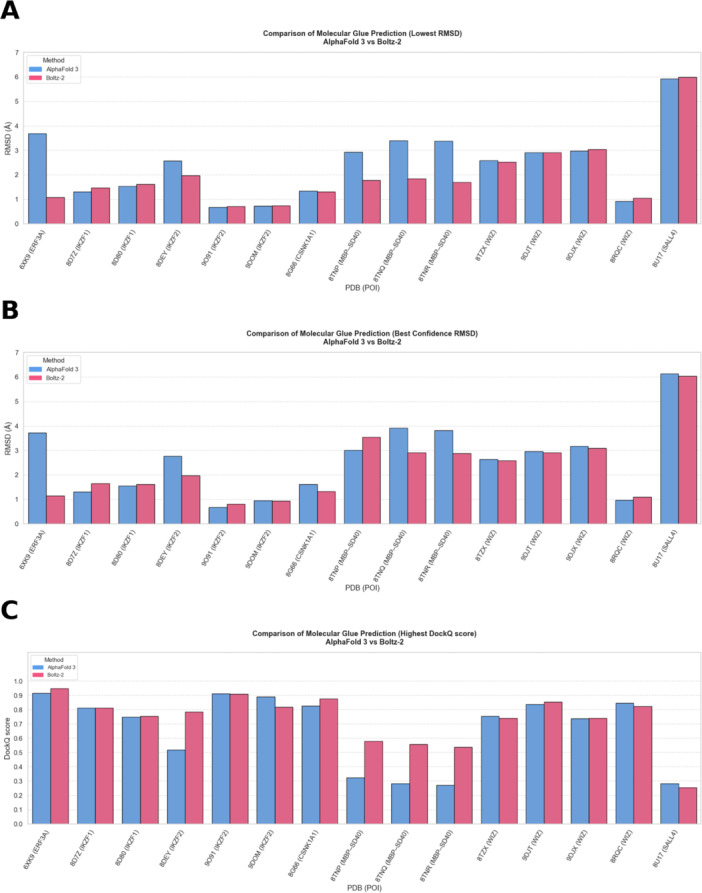
Comparison of AlphaFold 3 and Boltz‐2 performance on molecular glue‐mediated ternary complex predictions. Complex Cα‐RMSD is calculated relative to the experimental reference structure. (A) Minimum RMSD across all diffusion samples. (B) RMSD of the top‐ranked prediction according to the internal model confidence score. (C) Maximum DockQ score across all diffusion samples for each complex, evaluated against the experimental reference structure. RMSD, root‐mean‐square deviation.

The prediction of five samples of the molecular glue‐mediated ternary complexes required approximately 10–20 min using AlphaFold 3 and 2–7 min using Boltz‐2. Both models achieved good predictive accuracy, with most complex Cα‐RMSD values ranging from approximately 0.75–3 Å (Figure 3). Boltz‐2 produced overall slightly lower RMSD values. Larger performance differences were observed for the ternary complexes 6XK9, 8TNP, 8TNQ, and 8TNR, where AlphaFold 3 underperformed compared to Boltz‐2. For most of the remaining complexes, both models show comparable results. DockQ‐based evaluation was generally consistent with the RMSD trends. Differences between the models were overall smaller than those observed for PROTAC‐mediated ternary complexes, indicating comparable interface prediction performance. Notably, for 6XK9, AlphaFold 3 exhibited a higher RMSD than Boltz‐2, whereas the corresponding DockQ scores were similar, suggesting that the interface was largely preserved despite greater global deviation.

**Figure 4:**
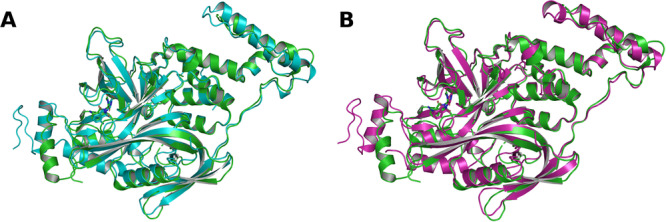
Superposition of molecular glue‐mediated ternary complex predictions for PDB ID 8U17. Panels show the lowest‐RMSD predictions for each model overlaid onto the experimental reference structure. (A) AlphaFold 3 prediction. (B) Boltz‐2 prediction. SALL4 (POI) is shown on the left and CRBN (E3 ligase) on the right. Proteins are represented as cartoons and the molecular glue ligand as sticks. The experimental reference structure is shown in green, the AlphaFold 3 prediction in cyan, and the Boltz‐2 prediction in pink. The corresponding complex RMSD values are 5.92 Å for AlphaFold 3 and 5.98 Å for Boltz‐2. CRBN, cereblon; POI, proteins of interest; RMSD, root‐mean‐square deviation.

When the top‐ranked predictions according to the internal confidence score were evaluated, both models showed comparable performance, as the slightly increased RMSD values for Boltz‐2 in 8TNP, 8TNQ, and 8TNR reduced the previously observed difference between the models. Aside from these complexes, both models demonstrated relatively high consistency between confidence‐based ranking and structural accuracy. Both models show comparatively poor prediction accuracy for 8U17 (Figure 4). As SALL4 is relatively small, both AlphaFold 3 and Boltz‐2 predicted similar but incorrect global arrangements of the complex with SALL4, despite accurately modeling CRBN and the molecular glue ligand.

We analyzed the prediction accuracy in relation to the PDB release dates of the complexes and the training cutoffs of AlphaFold 3 (September 30, 2021) and Boltz‐2 (June 1, 2023) (Supporting Information: Figure 3). For PROTAC‐mediated ternary complexes, RMSD values show a tendency to increase for structures deposited after the respective training cutoffs. This observation is expected, as these structures were not available during model training and highlights the strong dependence of the prediction accuracy on PDB coverage. The number of structures released before the training cutoffs for contrast for molecular glue‐mediated ternary complexes is insufficient to assess meaningful trends.

### 2.3 Output Metrics

We next assessed the correlation between the different output metrics of AlphaFold 3 and Boltz‐2 and the calculated RMSD values.

**Figure 5:**
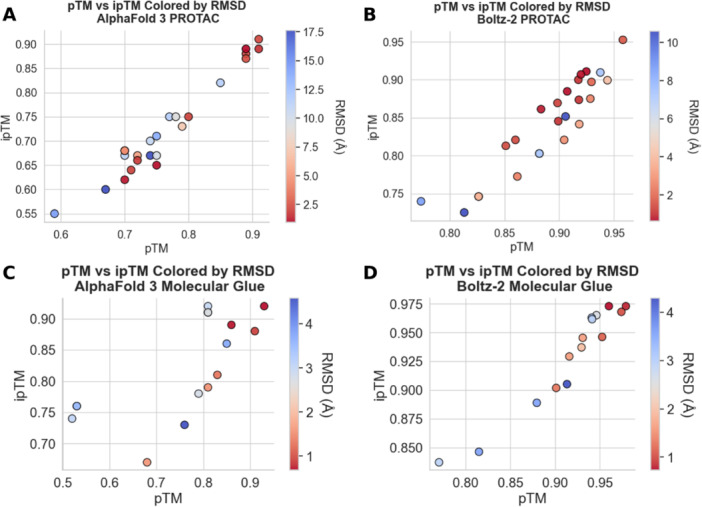
Relationship between prediction accuracy and model output metrics. (A) pTM versus ipTM for PROTAC ternary complex predictions by AlphaFold 3, colored by complex RMSD. (B) pTM versus ipTM for PROTAC ternary complex predictions by Boltz‐2, colored by complex RMSD. (C) pTM versus ipTM for molecular glue ternary complex predictions by AlphaFold 3, colored by complex RMSD. (D) pTM versus ipTM for molecular glue ternary complex predictions by Boltz‐2, colored by complex RMSD. PROTAC, proteolysis targeting chimeras; RMSD, root‐mean‐square deviation.

Both models show a weak to moderate negative correlation between complex RMSD and their output metrics, with substantial scatter and high *p*‐values (Supporting Information: Figure 1), indicating that these metrics do not reliably predict structural accuracy except partly in cases with very low pTM or ipTM scores (Figure 5). pTM and ipTM are model output metrics reported by AlphaFold 3 and Boltz‐2 that estimate the predicted global topology of the complex and the accuracy of inter‐chain interfaces. Similar trends are observed for molecular glue complex predictions. Here both models also show only weak negative correlations between RMSD and output metrics, and the metrics provide limited discrimination of prediction accuracy. Slightly reduced metric correlations for AlphaFold 3 are largely driven by complexes 8TNP and 8TNQ, which share the same POI and exhibit notably low pTM values despite relatively low RMSD, suggesting possible limitations in current training data for this system, although the number of currently available molecular glue complexes is insufficient for meaningful trend analysis.

### 2.4 Interface Geometry and Twisting

One fundamental problem with some PROTAC ternary complexes is the lack of interactions at the protein–protein interface. The interaction of proteins is facilitated by the PROTAC. Besides complexes that form extensive contacts, the overall complex conformation is heavily dependent on the PROTAC's conformation and flexibility, which leads to rotational freedom and twisting around the PROTAC linker [3].

**Figure 6:**
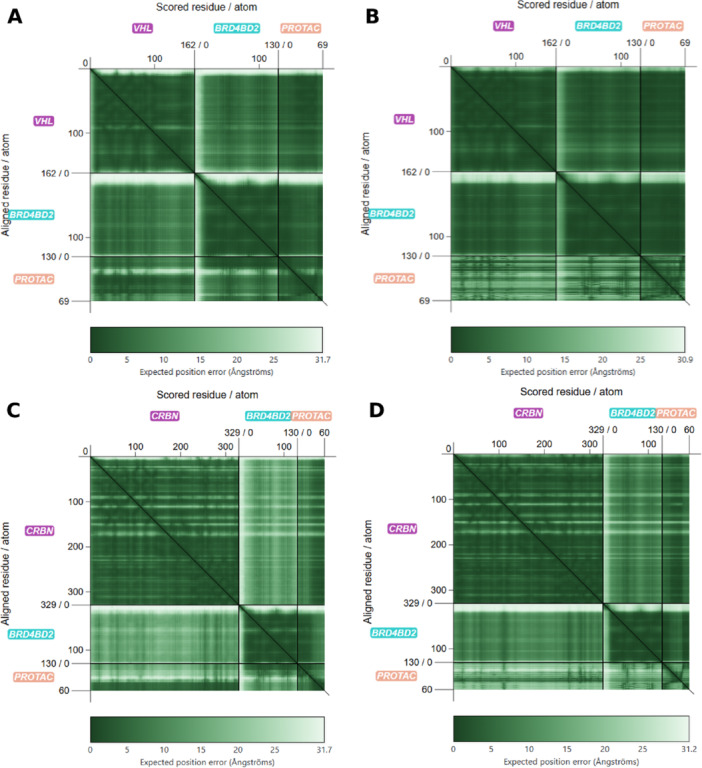
Predicted aligned error matrices for representative PROTAC ternary complexes. (A) AlphaFold 3 PAE matrix for the best predictions of PDB ID 8RQ9 (complex RMSD 6.99 Å). (B) Boltz‐2 PAE matrix for the best prediction of PDB ID 8RQ9 (complex RMSD 7.19 Å). (C) AlphaFold 3 PAE matrix for the best prediction of PDB ID 5T35 (complex RMSD 1.62 Å). (D) Boltz‐2 PAE matrix for the best prediction of PDB ID 5T35 (complex RMSD 0.58 Å). PAE, predicted aligned error; PDB, Protein Data Bank; PROTAC, proteolysis targeting chimeras; RMSD, root‐mean‐square deviation.

Examples of this behavior are the PROTAC ternary complex 8RQ9 and 5T35. The Predicted Aligned Error (PAE) matrices for the AlphaFold 3 and Boltz‐2 predictions indicate high predicted inter‐chain and ligand positional uncertainty (Figure 6). The corresponding predicted complexes also exhibit comparatively high RMSD values (6.99 Å for AlphaFold 3, 7.19 Å for Boltz‐2). In contrast, the PAE matrices of the 5T35 PROTAC ternary complex indicate higher positional certainty for both inter‐protein and ligand regions and the corresponding predictions show substantially lower RMSD values (1.62 Å for AlphaFold 3, 0.58 Å for Boltz‐2). Both 8RQ9 and 5T35 contain BRD4BD2 as the POI, but 8RQ9 recruits CRBN and 5T35 recruits VHL as the E3 ligase, which likely reflects differences in stability and geometry of their interfaces.

**Figure 7:**
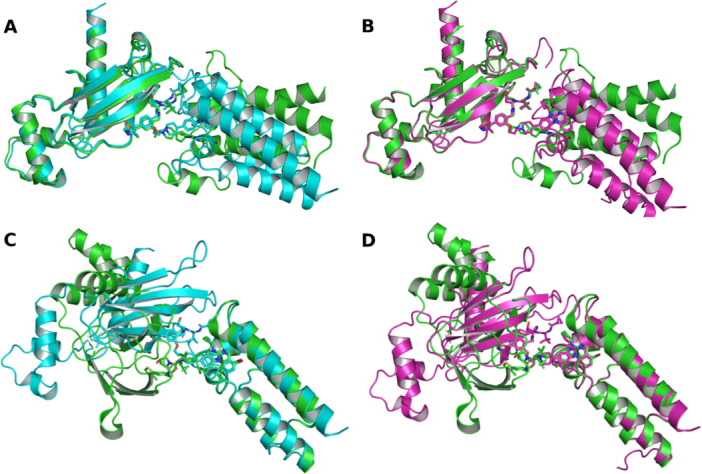
Rigid‐body superposition of PROTAC ternary complex predictions for PDB ID 7Z6L. All panels show the lowest RMSD predictions for each model, overlaid onto the experimental reference complex. Superposition is performed on the indicated protein, while the full ternary complex is displayed. (A) AlphaFold 3 prediction superposed on VHL. (B) Boltz‐2 prediction superposed on VHL. (C) AlphaFold 3 prediction superposed on the POI SMARCA2. (D) Boltz‐2 prediction superposed on SMARCA2. Proteins are shown as cartoons and PROTAC ligands as sticks. The reference crystal structure is shown in green, AlphaFold 3 predictions in cyan, and Boltz‐2 predictions in pink. The RMSD values relative to the reference structure are 0.97 Å (VHL), 0.96 Å (SMARCA2), and 9.60 Å (complex) for AlphaFold 3, and 0.99 Å (VHL), 0.92 Å (SMARCA2), and 14.76 Å (complex) for Boltz‐2. PDB, Protein Data Bank; PROTAC, proteolysis targeting chimeras; RMSD, root‐mean‐square deviation; VHL, von Hippel‐Lindau.

In several predictions the POI and E3 ligase are individually modeled with high structural accuracy, while their relative orientation within the ternary complex is incorrect (Figure 7). This is illustrated by the complex 7Z6L, containing VHL and SMARCA2, which exhibits large complex RMSD values (9.60 Å for AlphaFold 3 and 14.76 Å for Boltz‐2) and RMSD values below 1 Å for both SMARCA2 and VHL when evaluated individually. This rigid‐body rotational error is also not adequately captured by model output metrics such as pTM and ipTM, which remain high in these cases. This indicates that these metrics are largely insensitive to errors in the relative positioning of correctly folded subunits, which likely contributes to their weak correlation with complex RMSD and limits their usefulness for assessing global ternary complex assembly accuracy. Although reduced DockQ values indicate impaired interface quality, the metric does not fully capture global misorientation of correctly folded subunits. Consequently, the combined interpretation of DockQ and complex RMSD provides a more informative assessment of ternary complex assembly than either metric alone, particularly in cases involving twisting‐related deviations.

**Figure 8:**
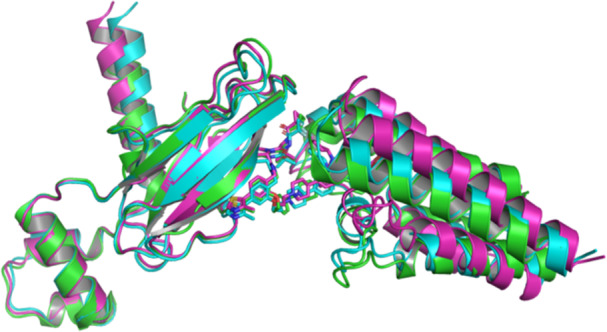
Superposition of the lowest RMSD predictions for PDB ID 7Z6L compared to the crystal structure of PDB ID 6HAX. The AlphaFold 3 prediction is shown in cyan, the Boltz‐2 prediction in pink, and the 6HAX reference structure in green. VHL is shown on the left and SMARCA2 on the right. Proteins are shown as cartoons and the PROTAC ligand as sticks. Both predictions adopt a ternary complex conformation similar to the one observed in the 6HAX crystal structure. PDB, Protein Data Bank; PROTAC, proteolysis targeting chimeras; RMSD, root‐mean‐square deviation.

A further limitation is observed for systems with similar proteins but different PROTACs (Figure 8). The PDB crystal structures for both 7Z6L and 6HAX have VHL as E3 ligase and SMARCA2 as POI, but different PROTACs. The crystal structures of both ternary complexes show two different conformations. AlphaFold 3 and Boltz‐2 predict a similar structure for 7Z6L as for 6HAX, which can also be found in the PDB crystal structures 6HAY and 6HR2. Predictions in this case could converge on protein‐protein interface orientations that resemble the ones frequently observed in the training data, even when the actual structure adopts a different geometry.

### 2.5 Limitations

Although Boltz‐2 outperformed AlphaFold 3 in terms of structural accuracy, as measured by RMSD, it is unclear whether this is due to differences in the recency of the training data or the model architecture. The later training cutoff of Boltz‐2 (June 1, 2023) includes various newer ternary complexes not present in the AlphaFold 3 (September 30, 2021) training data. This difference in training data is especially relevant for PROTAC and molecular glue complexes, as they are relatively new areas of research with structural data only beginning to accumulate in recent years. This complicates benchmarking, as many evaluated complexes are likely present in the model training data, making it difficult to interpret observed performance. There are not enough newly resolved complexes currently available to make fully independent benchmarking possible. We observed that the models perform significantly worse for complexes which appeared after the respective release dates. Most available structures are VHL‐, CRBN‐ and to a smaller extent cIAP1‐ and BIRC2‐based, which results in limited training diversity. For molecular glue ternary complexes, this is further pronounced, with even fewer structures available. The MD ensembles used in the training data of Boltz‐2 could have also improved the prediction quality. Having models trained on more comparable datasets would make a more direct assessment for comparing architectures possible.

In the predicted PROTAC complexes of both models, most errors stemmed from flexible, structurally unusual complexes and twisting or misaligned orientation around the linker. A similar study that used AlphaFold 3 for predicting PROTAC ternary complexes also observed this behavior [15]. CRBN‐based complexes were generally less accurately predicted than VHL‐based ones, possibly due to limited structural diversity in the training data. Intrinsic effects of the ligase complexes potentially also contribute such as differences in flexibility, binding interfaces, linker length and structural organization.

For molecular glue complexes, most errors stemmed from slight interface misplacement in structurally unusual complexes. Predicted molecular glue complexes generally showed higher structural accuracy than PROTACs, possibly due to their smaller size, lack of flexible linkers and simpler binding topology.

Even though these models provide a fast way to generate TPD complex structures, they do not replace crystallographic or cryo‐EM data and require experimental verification. The findings here reinforce that the strength of the current models may overstate their utility in real‐world applications and generalizability for both PROTACs and molecular glue development. We find that prediction failures, such as misoriented ternary complexes and low performance for structurally unusual complexes, can be partially traced to overfitting toward frequently observed structures or interface types in the training set. However, deviations from the crystal structure do not necessarily imply biological implausibility, especially for very flexible complexes, because static experimental crystal structures do not fully capture the conformational variability in solution [5].

Protein degradation mediated through PROTACs and molecular glues is also dependent on formation of the full E3 ligase assembly like the CRL2‐VHL complex or CRL4A‐CRBN complex and the spatial accessibility of lysine residues for ubiquitination [16]. Cooperative effects for formation of the complexes are also important for the degradation [5]. These aspects are not directly accounted for by the current methods. It is also a known issue that these models often disregard ligand stereochemistry like chirality, which is especially important for drug modeling, where correct stereochemistry can substantially affect binding and activity [17].

Complex RMSD is commonly used to evaluate prediction, but it strongly penalizes rigid‐body rotations and hinge shifts that may not disrupt functional binding interfaces. DockQ scores provide a complementary, interface‐sensitive assessment and were generally consistent with RMSD trends in our benchmark, however, they were insufficient to comprehensively capture errors attributable to global misorientation. Output metrics such as pTM and ipTM were more a reflection of internal consistency rather than correctness of the models, especially when protein‐protein interfaces are weak. In this work, the predictions with the highest confidence score were often not the ones with the lowest RMSD, although they were generally close. These cases show that manual clustering of the predicted samples remains necessary.

## 3 Conclusion

In this work, we benchmarked the AI‐based structure prediction models AlphaFold 3 and Boltz‐2 for the prediction of ternary complexes of PROTACs and molecular glues. Both models achieved reasonably high accuracy for most tested complexes and surpassed existing methods in both speed and predictive accuracy. Most errors stemmed from flexible, structurally unusual complexes and twisting or misaligned orientation around the linker, which is partially expected for models trained on static PDB data and should be taken into account when using them. Performance was generally lower for CRBN‐based PROTAC complexes than for VHL‐based ones, likely because of differences in training data, although intrinsic effects of the ligase complexes may also contribute. For the tested CRBN molecular glue complexes, the accuracy in comparison to PROTACs was higher with most errors stemming from slight interface misplacement in structurally unusual complexes. Boltz‐2 slightly outperformed AlphaFold 3 for both PROTAC and molecular glue complexes. Reasons for this could be both in the architecture or training data, but the extent to which each contributes is unclear. The fully open‐source structure of Boltz‐2 also offers more potential for modifications. Future additional structural data could improve the performance and generalization of retrained or new model versions and would also make it possible to extend the applicability to other E3 ligases.

## 4 Materials and Methods

### 4.1 Model Setup and Input Preparation

AlphaFold 3 was installed locally with Docker following the official GitHub installation instructions [12] and executed on a PC with an NVIDIA GeForce RTX 4070 GPU and NVIDIA CUDA 12.6. Boltz‐2 predictions were performed in a cloud environment using Google Colab Pro with an NVIDIA L4 GPU and NVIDIA CUDA 12.6 through a Jupyter notebook, following the official GitHub instructions [13]. A Google Colab Jupyter notebook implementing the Boltz‐2 workflow is available in the Data Availability section. Boltz‐2 was additionally installed on the same system used for AlphaFold 3 to compare differences in prediction speed, which showed similar results to the cloud environment.

Aside from E3 ligase, POI, and PROTAC/molecular glue, any accessory components, like Elongin B/C, DNA damage‐binding protein 1, further ligands or solvent molecules were removed from the complexes in the final dataset. All ligands were used as canonical SMILES produced by OpenEye OEToolkit 1.7.6.

To ensure comparability between the methods, AlphaFold 3 and Boltz‐2 were run with the same model input configuration parameters (Table 3). The Input file formats were used according to the specifications of each model. Each prediction was run with five diffusion samples.

**Table 3:** Model input configuration for AlphaFold 3 and Boltz‐2 used in this study.

Input parameter	Description
**File format** AlphaFold 3: JSON Boltz‐2: YAML	JSON for AlphaFold 3 and YAML for Boltz‐2, each including the protein FASTA sequences, ligand SMILES and additional configuration parameters.
**MSA generation** AlphaFold 3: JackHMMer Boltz‐2: MMseqs. 2	AlphaFold 3 uses the sequence search program JackHMMer for multiple sequence alignment generation and Boltz‐2 uses MMseqs. 2.
**Recycling steps** configuration: 10	Repeated reprocessing of the predicted coordinates within the model.
**Diffusion sampling steps** configuration: 200	Number of denoising steps during the diffusion process.
**Diffusion samples** configuration: 5	Number of independent diffusion trajectories generated, corresponding to the number of output structures.
**Seed** configuration: 1	Fixed seed for reproducibility of the stochastic sampling.

Preliminary tests showed that stochastic variation across independent seeds produced only minor deviations in RMSD and structure. Consequently, to maintain reproducibility and minimize computational cost, a single fixed stochastic seed was used for all predictions.

### 4.2 Evaluation and Validation Metrics

Structural deviations were quantified as complex Cα‐RMSD using MOE 2019.01. For the calculation, each ternary complex (E3 ligase, ligand, and POI) was superposed as a single rigid body onto the corresponding reference PDB structure to preserve the relative orientation of chains and the overall interface geometry. DockQ scores were calculated using DockQ v2 as described by Mirabello and Wallner [18]. DockQ v2 integrates interface RMSD, ligand RMSD, and the fraction of native contacts into a single continuous score ranging from 0 to 1 and was computed relative to the corresponding experimental reference structures. In addition, prediction quality was evaluated using output metrics of the models. In both models several of these metrics are combined into a single numerical value used to rank output structures. AlphaFold refers to this metric as ranking score, whereas Boltz‐2 refers to it as confidence score. The underlying calculations differ between the models (Table 4) For clarity, we refer to both as confidence scores throughout this work. Correlations between model output metrics (pTM, ipTM, and confidence score) and RMSD were quantified with Pearson correlation to evaluate their relationship to structural accuracy and each other (Supporting Information: Figure 1).

**Table 4:** Model output metrics reported by AlphaFold 3 and Boltz‐2.

Output metric	Description
pTM (predicted TM‐score)	pTM estimates the global topological accuracy of the complex, scaled from 0 to 1.
ipTM (interface pTM‐score)	ipTM estimates the predicted accuracy of all inter‐chain interfaces in the complex, scaled from 0 to 1.
pLDDT (predicted local distance difference test)	Local score reported per residue for proteins and per atom for ligands, reflecting the model confidence in the spatial placement relative to the surrounding structure, scaled from 0 to 100.
PAE (Predicted Aligned Error)	Residue‐residue matrix of expected positional deviations after structural alignment. Lower values indicate more certain orientation.
Ranking/confidence score	AlphaFold 3 ranking score (1): $0.8\times \mathrm{ipTM}+0.2\times \mathrm{pTM}+0.5\times f_{\mathrm{dis}}-100\times C_{\mathrm{clash}}$ $f_{\mathrm{dis}}$ estimates unresolved regions, scaled from 0 to 1 $C_{\mathrm{clash}}$ is a boolean for atomic clashes when more than 50% of a chain or over 100 atoms are involved Boltz‐2 confidence score (2): $0.8\times \bar{pLDDT}+0.2\times \mathrm{ipTM}$ The ranking/confidence score is a single numerical value used to rank predictions, higher values suggest better predictions, scaled from 0 to 1.

Each model generated five output structures per prediction. These structures were evaluated both by their Cα‐RMSD relative to the experimental reference PDB structure and by their confidence scores. This was done because in practical scenarios, reference structures are generally not available, so aside from structure, models mostly need to be ranked by intrinsic metrics. For each of the predicted complexes, binding geometry, ligand placement and ligand interactions were also visually inspected and compared to the experimental complexes using Schrödinger Maestro 2021 and 2025.

## Author Contributions

**Lino Riepenhausen:** investigation, methodology, software, data curation, formal analysis, visualization, writing – original draft. **Anne‐C. Sarnow:** methodology, software, data curation. **Dina Robaa:** writing – review and editing. **Wolfgang Sippl:** conceptualization, data curation, resources, supervision, writing – review and editing.

## Conflicts of Interest

The authors declare no conflicts of interest.

## Supporting information


**Figure 1:** Correlations between lowest complex RMSD and model output metrics for predicted ternary complexes (pTM, ipTM and confidence score) for PROTAC‐mediated ternary complexes predicted by AlphaFold 3 (A) and Boltz‐2 (B) and for molecular glue‐mediated ternary complexes predicted by AlphaFold 3 (C) and Boltz‐2 (D). Pearson correlation coefficients and corresponding p values are reported in each panel. **Figure 2:** AlphaFold 3 and Boltz‐2 RMSD performance comparison for HADDOCK, ICM, MOE method 4B and PRosettaC PROTAC ternary complexes from the benchmark study (4). Only complexes that are both present in this work and the benchmark study are shown. For all methods, the lowest C ‐RMSD for predicted clusters are compared. **Figure 3:** Prediction accuracy relative to PDB release date and model training cutoffs. (A) Complex RMSD of PROTAC‐mediated ternary complex predictions plotted against PDB release date. (B) Complex RMSD of molecular glue‐mediated complex predictions plotted against PDB release date. Vertical dashed lines indicate the training data cutoffs of AlphaFold 3 (30 September 2021) and Boltz‐2 (1 June 2023). **Table 1:** Overview of PDB complexes used for benchmarking. **Table 2:** AlphaFold 3 and Boltz‐2 Output metrics and RMSD for PROTAC complexes. Column RMSD is the lowest RMSD of the five complexes and Cfd RMSD is the RMSD of the complex with the highest confidence score. DockQ Score is the highest DockQ score of the five complexes calculated with DockQ v2.

## Data Availability

Data for all PROTAC and molecular glue complexes used in this study, including RMSD values and additional evaluation metrics, are provided in Appendix. A Google Colab Jupyter notebook for Boltz‐2 ternary complex structure prediction is available on GitHub at https://gist.github.com/linoriep/ead7b3f02be04404b7d930ad497954c7.
