# Supplementary material for: AI‐Based Prediction of PROTAC‐ and Molecular Glue‐Mediated Ternary Complexes: A Comparative Evaluation of AlphaFold 3 and Boltz‐2
Source: Arch Pharm (Weinheim). 2026 Mar 14;359(3):e70225. doi: 10.1002/ardp.70225 (PMC12988791; doi:10.1002/ardp.70225)
Supplement: Supplementary file 1 — Figure 1: Correlations between lowest complex RMSD and model output metrics for predicted ternary complexes (pTM, ipTM and confidence score) for PROTAC‐mediated ternary complexes predicted by AlphaFold 3 (A) and Boltz‐2 (B) and for molecular glue‐mediated ternary complexes predicted by AlphaFold 3 (C) and Boltz‐2 (D). Pearson correlation coefficients and corresponding p values are reported in each panel. Figure 2: AlphaFold 3 and Boltz‐2 RMSD performance comparison for HADDOCK, ICM, MOE method 4B and PRosettaC PROTAC ternary complexes from the benchmark study (4). Only complexes that are both present in this work and the benchmark study are shown. For all methods, the lowest C ‐RMSD for predicted clusters are compared. Figure 3: Prediction accuracy relative to PDB release date and model training cutoffs. (A) Complex RMSD of PROTAC‐mediated ternary complex predictions plotted against PDB release date. (B) Complex RMSD of molecular glue‐mediated complex predictions plotted against PDB release date. Vertical dashed lines indicate the training data cutoffs of AlphaFold 3 (30 September 2021) and Boltz‐2 (1 June 2023). Table 1: Overview of PDB complexes used for benchmarking. Table 2: AlphaFold 3 and Boltz‐2 Output metrics and RMSD for PROTAC complexes. Column RMSD is the lowest RMSD of the five complexes and Cfd RMSD is the RMSD of the complex with the highest confidence score. DockQ Score is the highest DockQ score of the five complexes calculated with DockQ v2. [file ARDP-359-e70225-s001.docx]

**Supporting Information**

**AI-Based Prediction of PROTAC- and Molecular Glue-Mediated Ternary Complexes: A Comparative Evaluation of AlphaFold 3 and Boltz-2**

Lino Riepenhausen^1^, Anne-Christin Sarnow^1^, Dina Robaa^1^, Wolfgang Sippl^1^

^1^Department Medicinal Chemistry, Institute of Pharmacy

Martin-Luther-Universität Halle-Wittenberg

06120 Halle/Saale, Germany

# *
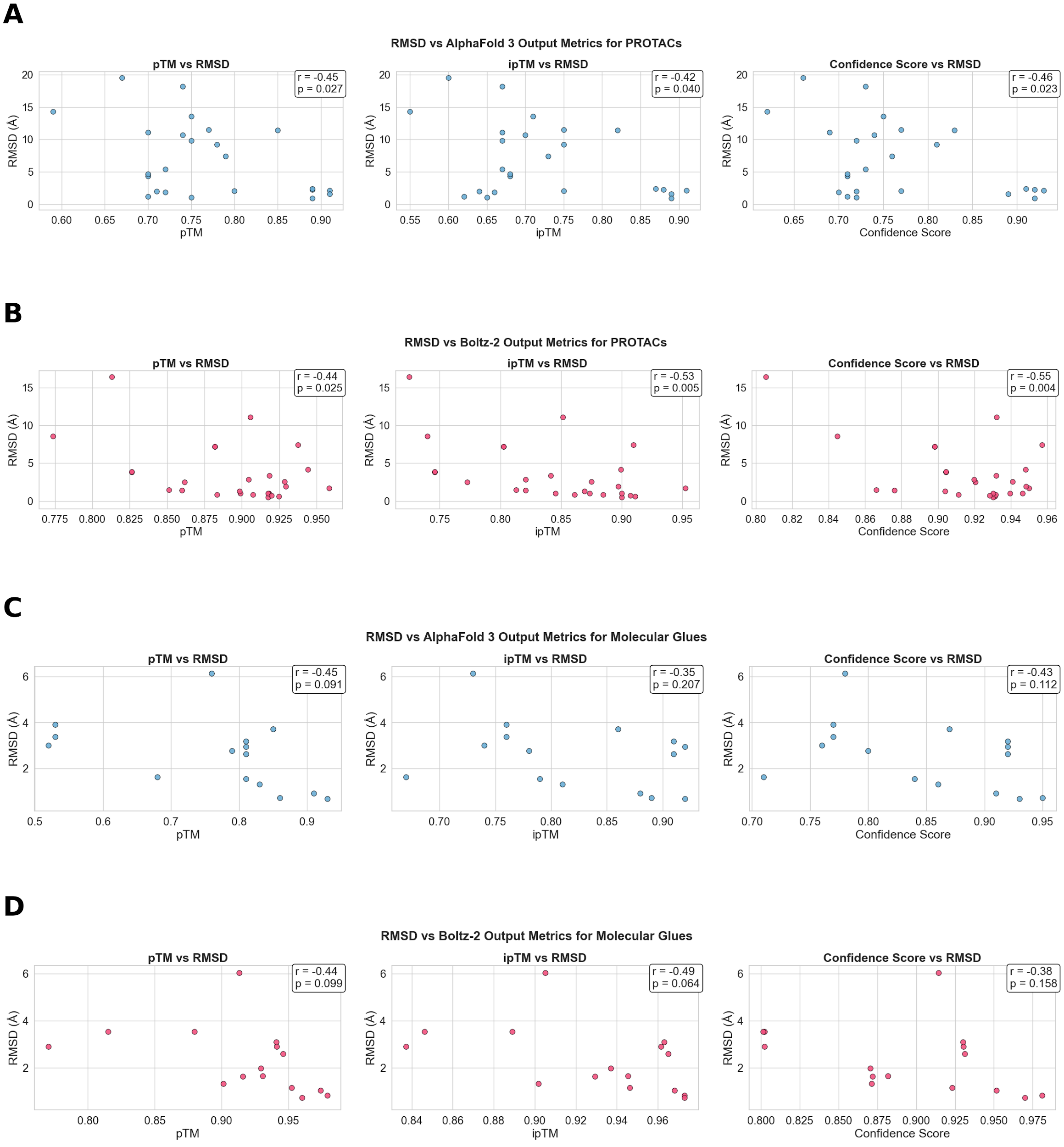
*

***Figure 1:*** *Correlations between lowest complex RMSD and model output metrics for predicted ternary complexes (pTM, ipTM and confidence score) for PROTAC-mediated ternary complexes predicted by AlphaFold 3 (A) and Boltz-2 (B) and for molecular glue-mediated ternary complexes predicted by AlphaFold 3 (C) and Boltz-2 (D). Pearson correlation coefficients and corresponding p values are reported in each panel.*


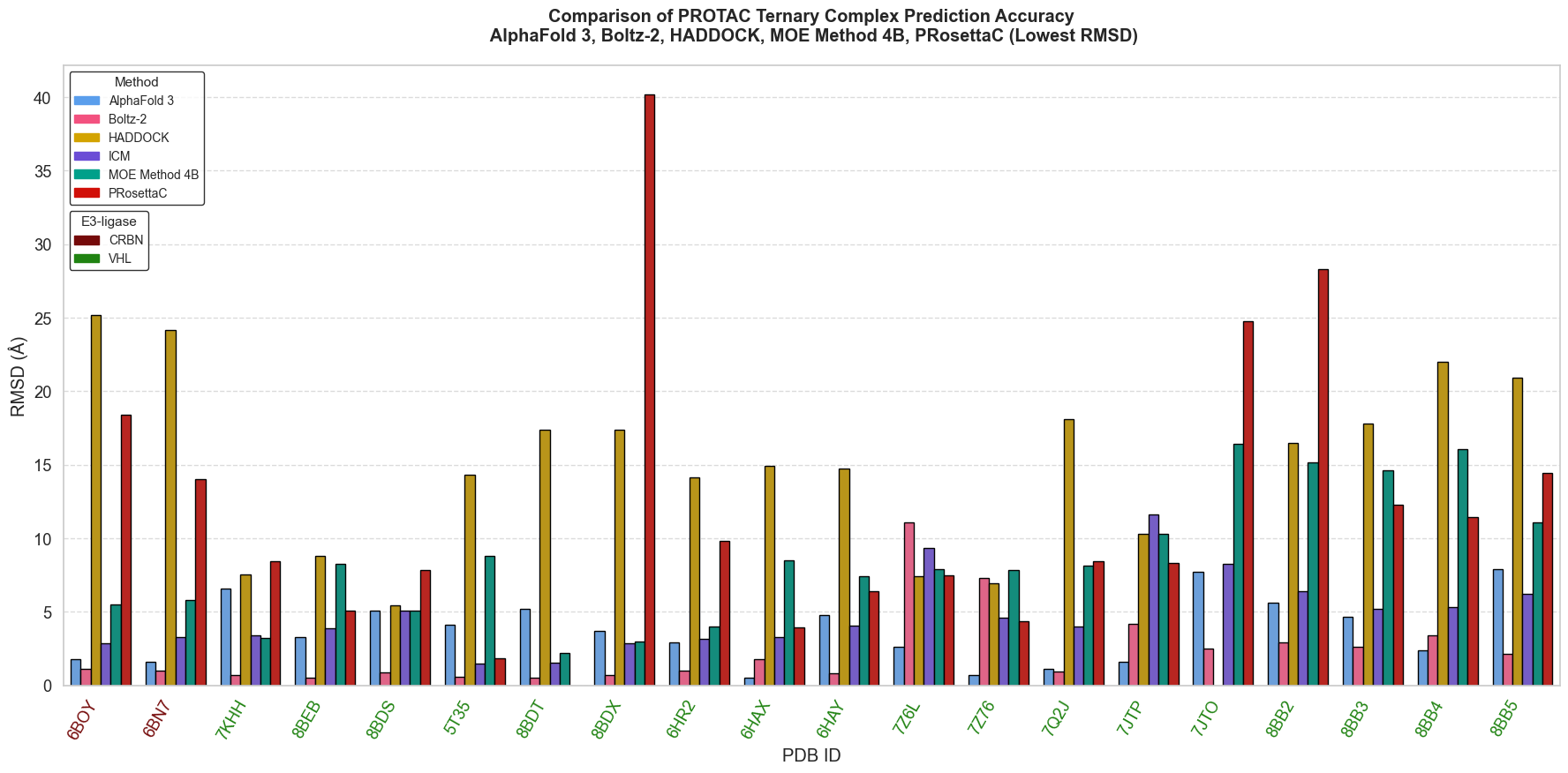


***Figure 2:*** *AlphaFold 3 and Boltz-2 RMSD performance comparison for HADDOCK, ICM, MOE method 4B and PRosettaC PROTAC ternary complexes from the benchmark study (*[4)](https://www.zotero.org/google-docs/?tvGv64)*. Only complexes that are both present in this work and the benchmark study are shown. For all methods, the lowest C*$\alpha$*-RMSD for predicted clusters are compared.*


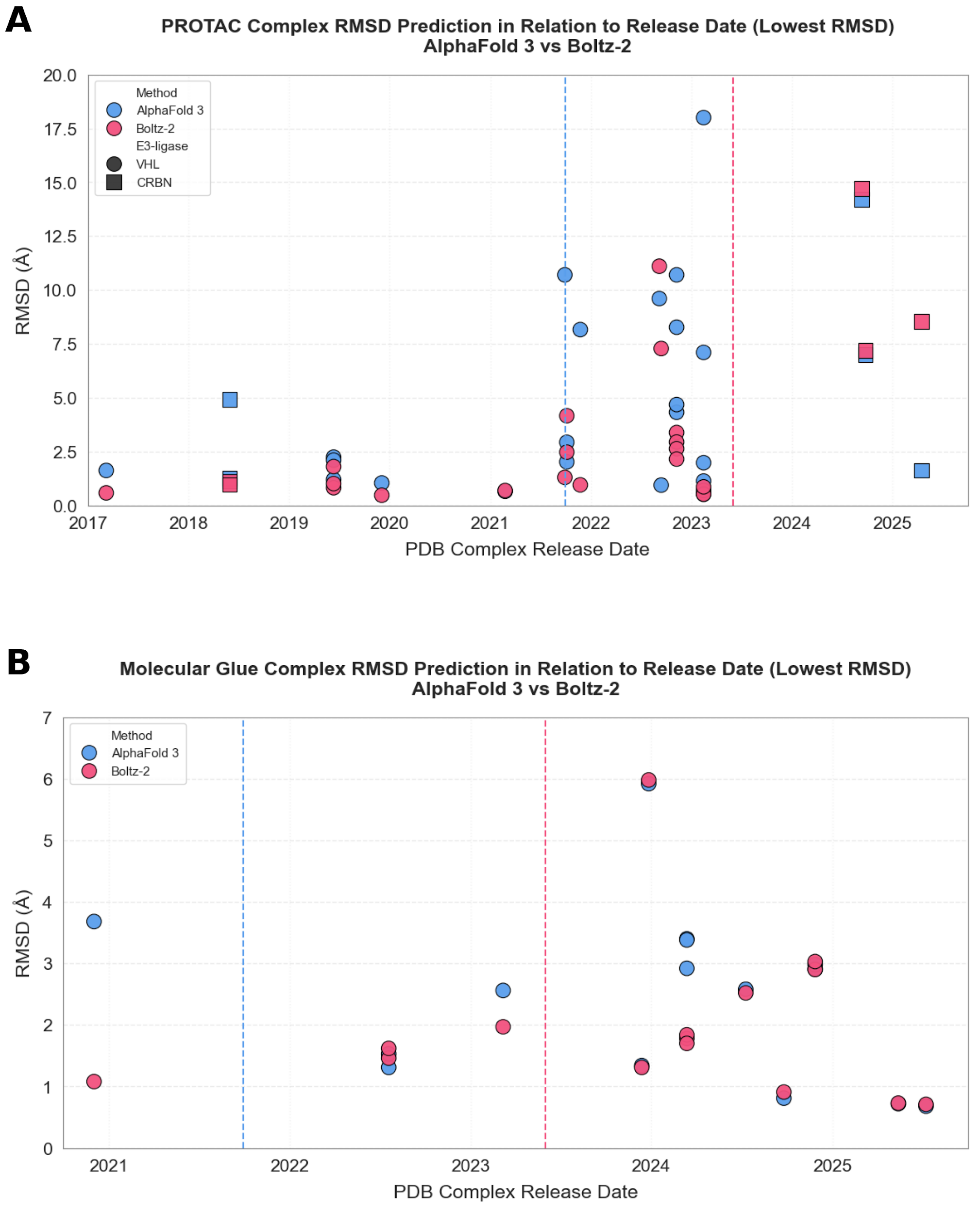


***Figure 3:*** *Prediction accuracy relative to PDB release date and model training cutoffs. (A) Complex RMSD of PROTAC-mediated ternary complex predictions plotted against PDB release date. (B) Complex RMSD of molecular glue-mediated complex predictions plotted against PDB release date. Vertical dashed lines indicate the training data cutoffs of AlphaFold 3 (30 September 2021) and Boltz-2 (1 June 2023).*

***Table 1:*** *Overview of PDB complexes used for benchmarking.*

| **PDB ID** | **E3 Ligase** | **POI** | **Ligand** | **Release Date** | **Resolution (Å)** |
| --- | --- | --- | --- | --- | --- |
|  | | |  |  |  |
| 5T35 | VHL | BRD4${}^{BD2}$ | MZ1 | 2017-03-08 | 2.70 |
| 6BOY | CRBN | BRD4${}^{BD1}$ | RN6 | 2018-05-30 | 3.33 |
| 6BN7 | CRBN | BRD4${}^{BD1}$ | RN3 | 2018-05-30 | 3.50 |
| 6HAY | VHL | SMARCA2 | FX8 | 2019-06-12 | 2.24 |
| 6HAX | VHL | SMARCA2 | FWZ | 2019-06-12 | 2.35 |
| 6HR2 | VHL | SMARCA4 | FWZ | 2019-06-12 | 1.76 |
| 6SIS | VHL | BRD4${}^{BD2}$ | LFE | 2019-12-04 | 3.50 |
| 7KHH | VHL | BRD4${}^{BD1}$ | WEP | 2021-02-24 | 2.28 |
| 7PI4 | VHL | FAK | 7QB | 2021-09-29 | 2.24 |
| 7JTP | VHL | WDR5 | X6M | 2021-10-06 | 2.12 |
| 7JTO | VHL | WDR5 | VKA | 2021-10-06 | 1.70 |
| 7Q2J | VHL | WDR5 | 8KH | 2021-11-24 | 2.50 |
| 7Z6L | VHL | SMARCA2 | IEI | 2022-09-07 | 2.24 |
| 7Z76 | VHL | SMARCA2 | IEJ | 2022-09-14 | 1.32 |
| 8BB2 | VHL | WDR5 | Q3X | 2022-11-09 | 2.05 |
| 8BB3 | VHL | WDR5 | Q3X | 2022-11-09 | 1.80 |
| 8BB4 | VHL | WDR5 | Q3R | 2022-11-09 | 2.80 |
| 8BB5 | VHL | WDR5 | Q43 | 2022-11-09 | 2.20 |
| 8BDS | VHL | BRD4${}^{BD1}$ | QIY | 2023-02-15 | 1.72 |
| 8BDT | VHL | BRD4${}^{BD2}$ | QLX | 2023-02-15 | 2.70 |
| 8BDX | VHL | BRD4${}^{BD2}$ | QIY | 2023-02-15 | 2.93 |
| 8BEB | VHL | BRD4${}^{BD1}$ | QIK | 2023-02-15 | 3.18 |
| 8RQ9 | VHL | BRD4${}^{BD2}$ | A1H2F | 2024-09-25 | 2.91 |
| 8UH6 | CRBN | PTPN2 | WO8 | 2024-09-11 | 3.30 |
| 9D0W | CRBN | CDK2 | A1LY0 | 2025-04-16 | 2.95 |
| **Molecular Glue Ternary Complexes** | | |  |  |  |
| 6XK9 | CRBN | GSPT1 | V4M | 2020-12-02 | 3.64 |
| 8D7Z | CRBN | IKZF1 | QFZ | 2022-07-20 | 3.10 |
| 8D80 | CRBN | IKZF1 | 8W7 | 2022-07-20 | 3.60 |
| 8DEY | CRBN | IKZF2 | LWK | 2023-03-08 | 3.70 |
| 8G66 | CRBN | CSNK1A1 | YOT | 2023-12-13 | 3.45 |
| 8TNP | CRBN | MBP–SD40 | Y70 | 2024-03-13 | 3.30 |
| 8TNQ | CRBN | MBP–SD40 | MIQ | 2024-03-13 | 2.41 |
| 8TNR | CRBN | MBP–SD40 | MIQ | 2024-03-13 | 2.50 |
| 8TZX | CRBN | WIZ | U3I | 2024-07-10 | 3.15 |
| 8U17 | CRBN | SALL4 | Y70 | 2023-12-27 | 3.10 |
| 9DJT | CRBN | WIZ | A1A5H | 2024-11-27 | 2.95 |
| 9DJX | CRBN | WIZ | A1A5I | 2024-11-27 | 3.35 |
| 9O91 | CRBN | IKZF2 | A1CAC | 2025-07-09 | 1.86 |
| 9DOM | CRBN | IKZF2 | A1A8N | 2025-05-14 | 1.69 |
| 8RQC | CRBN | WIZ | QFC | 2024-09-25 | 2.15 |

***Table 2:*** *AlphaFold 3 and Boltz-2 Output metrics and RMSD for PROTAC complexes. Column RMSD is the lowest RMSD of the five complexes and Cfd RMSD is the RMSD of the complex with the highest confidence score. DockQ Score is the highest DockQ score of the five complexes calculated with DockQ v2.*

| **PDB ID** | **Model** | **RMSD (Å)** | **Cfd**  **RMSD (Å)** | **Cfd** | **pTM** | **ipTM** | **DockQ score** |
| --- | --- | --- | --- | --- | --- | --- | --- |
| **PROTAC Ternary Complexes** | | | | | | | |
| 5T35 | AlphaFold 3 | 1.62 | 2.60 | 0.82 | 0.83 | 0.77 | 0.746 |
| 5T35 | Boltz-2 | 0.58 | 0.62 | 0.93 | 0.92 | 0.91 | 0.951 |
| 6BOY | AlphaFold 3 | 1.26 | 2.00 | 0.72 | 0.71 | 0.64 | 0.721 |
| 6BOY | Boltz-2 | 1.12 | 1.41 | 0.88 | 0.86 | 0.82 | 0.78 |
| 6BN7 | AlphaFold 3 | 4.92 | 5.40 | 0.73 | 0.72 | 0.67 | 0.299 |
| 6BN7 | Boltz-2 | 0.97 | 1.45 | 0.87 | 0.85 | 0.81 | 0.677 |
| 6HAX | AlphaFold 3 | 2.10 | 2.26 | 0.92 | 0.89 | 0.88 | 0.727 |
| 6HAX | Boltz-2 | 1.80 | 1.92 | 0.95 | 0.93 | 0.90 | 0.889 |
| 6HAY | AlphaFold 3 | 2.24 | 2.41 | 0.91 | 0.89 | 0.87 | 0.708 |
| 6HAY | Boltz-2 | 0.82 | 1.01 | 0.94 | 0.92 | 0.87 | 0.897 |
| 6HR2 | AlphaFold 3 | 1.20 | 1.59 | 0.89 | 0.91 | 0.89 | 0.819 |
| 6HR2 | Boltz-2 | 1.01 | 1.01 | 0.95 | 0.90 | 0.85 | 0.943 |
| 6SIS | AlphaFold 3 | 1.04 | 1.04 | 0.72 | 0.75 | 0.65 | 0.759 |
| 6SIS | Boltz-2 | 0.47 | 0.73 | 0.93 | 0.92 | 0.91 | 0.952 |
| 7KHH | AlphaFold 3 | 0.65 | 1.20 | 0.71 | 0.70 | 0.62 | 0.919 |
| 7KHH | Boltz-2 | 0.69 | 0.81 | 0.91 | 0.88 | 0.86 | 0.959 |
| 7PI4 | AlphaFold 3 | 10.70 | 11.40 | 0.83 | 0.85 | 0.82 | 0.108 |
| 7PI4 | Boltz-2 | 1.30 | 2.54 | 0.94 | 0.93 | 0.88 | 0.725 |
| 7JTP | AlphaFold 3 | 2.02 | 2.02 | 0.77 | 0.80 | 0.75 | 0.544 |
| 7JTP | Boltz-2 | 4.16 | 4.16 | 0.95 | 0.94 | 0.90 | 0.267 |
| 7JTO | AlphaFold 3 | 2.93 | 11.10 | 0.69 | 0.70 | 0.67 | 0.159 |
| 7JTO | Boltz-2 | 2.47 | 2.47 | 0.92 | 0.86 | 0.77 | 0.56 |
| 7Q2J | AlphaFold 3 | 8.16 | 9.22 | 0.81 | 0.78 | 0.75 | 0.107 |
| 7Q2J | Boltz-2 | 0.95 | 1.28 | 0.90 | 0.90 | 0.87 | 0.791 |
| 7Z6L | AlphaFold 3 | 9.60 | 9.82 | 0.72 | 0.75 | 0.67 | 0.141 |
| 7Z6L | Boltz-2 | 11.10 | 11.10 | 0.93 | 0.91 | 0.85 | 0.061 |
| 7Z76 | AlphaFold 3 | 0.94 | 0.94 | 0.92 | 0.89 | 0.89 | 0.913 |
| 7Z76 | Boltz-2 | 7.28 | 7.40 | 0.96 | 0.94 | 0.91 | 0.033 |
| 8BB2 | AlphaFold 3 | 4.32 | 4.32 | 0.71 | 0.70 | 0.68 | 0.141 |
| 8BB2 | Boltz-2 | 2.94 | 3.80 | 0.90 | 0.83 | 0.75 | 0.461 |
| 8BB3 | AlphaFold 3 | 4.68 | 4.68 | 0.71 | 0.70 | 0.68 | 0.179 |
| 8BB3 | Boltz-2 | 2.63 | 3.87 | 0.90 | 0.83 | 0.75 | 0.654 |
| 8BDS | AlphaFold 3 | 1.13 | 1.26 | 0.87 | 0.87 | 0.85 | 0.885 |
| 8BDS | Boltz-2 | 0.69 | 1.02 | 0.93 | 0.92 | 0.90 | 0.974 |
| 8BEB | AlphaFold 3 | 1.98 | 2.13 | 0.93 | 0.91 | 0.91 | 0.696 |
| 8BEB | Boltz-2 | 0.52 | 1.70 | 0.95 | 0.96 | 0.95 | 0.916 |
| 8BDT | AlphaFold 3 | 18.00 | 18.20 | 0.73 | 0.74 | 0.67 | 0.019 |
| 8BDT | Boltz-2 | 0.52 | 0.52 | 0.93 | 0.92 | 0.90 | 0.946 |
| 8BDX | AlphaFold 3 | 7.10 | 19.50 | 0.66 | 0.67 | 0.60 | 0.049 |
| 8BDX | Boltz-2 | 0.69 | 0.85 | 0.93 | 0.91 | 0.88 | 0.937 |
| 8RQ9 | AlphaFold 3 | 6.99 | 7.40 | 0.76 | 0.79 | 0.73 | 0.35 |
| 8RQ9 | Boltz-2 | 7.19 | 7.19 | 0.90 | 0.88 | 0.80 | 0.099 |
| 8UH6 | AlphaFold 3 | 14.20 | 14.30 | 0.62 | 0.59 | 0.55 | 0.025 |
| 8UH6 | Boltz-2 | 14.70 | 16.40 | 0.81 | 0.81 | 0.73 | 0.035 |
| 9D0W | AlphaFold 3 | 1.62 | 3.23 | 0.67 | 0.73 | 0.68 | 0.442 |
| 9D0W | Boltz-2 | 8.55 | 8.59 | 0.84 | 0.77 | 0.74 | 0.221 |

| **PDB ID** | **Model** | **RMSD (Å)** | **Cfd**  **RMSD (Å)** | | **Cfd** | **pTM** | **ipTM** | **DockQ score** |
| --- | --- | --- | --- | --- | --- | --- | --- | --- |
| **Molecular Glue Ternary Complexes** | | | | |  |  |  |  |
| 6XK9 | AlphaFold 3 | 3.68 | 3.71 | 0.87 | | 0.85 | 0.86 | 0.86 |
| 6XK9 | Boltz-2 | 1.08 | 1.15 | 0.92 | | 0.95 | 0.95 | 0.95 |
| 8D7Z | AlphaFold 3 | 1.31 | 1.31 | 0.86 | | 0.83 | 0.81 | 0.81 |
| 8D7Z | Boltz-2 | 1.46 | 1.65 | 0.88 | | 0.93 | 0.95 | 0.95 |
| 8D80 | AlphaFold 3 | 1.53 | 1.55 | 0.84 | | 0.81 | 0.79 | 0.79 |
| 8D80 | Boltz-2 | 1.62 | 1.62 | 0.87 | | 0.92 | 0.93 | 0.93 |
| 8DEY | AlphaFold 3 | 2.56 | 2.76 | 0.80 | | 0.79 | 0.78 | 0.78 |
| 8DEY | Boltz-2 | 1.97 | 1.97 | 0.87 | | 0.93 | 0.94 | 0.94 |
| 8G66 | AlphaFold 3 | 1.34 | 1.62 | 0.71 | | 0.68 | 0.67 | 0.324 |
| 8G66 | Boltz-2 | 1.31 | 1.32 | 0.87 | | 0.90 | 0.90 | 0.579 |
| 8TNP | AlphaFold 3 | 2.92 | 2.92 | 0.76 | | 0.53 | 0.74 | 0.282 |
| 8TNP | Boltz-2 | 1.78 | 3.54 | 0.80 | | 0.88 | 0.89 | 0.558 |
| 8TNQ | AlphaFold 3 | 3.40 | 3.40 | 0.77 | | 0.53 | 0.76 | 0.271 |
| 8TNQ | Boltz-2 | 1.84 | 2.90 | 0.80 | | 0.77 | 0.84 | 0.537 |
| 8TNR | AlphaFold 3 | 3.38 | 3.81 | 0.77 | | 0.53 | 0.76 | 0.271 |
| 8TNR | Boltz-2 | 1.7 | 2.87 | 0.80 | | 0.77 | 0.84 | 0.537 |
| 8TZX | AlphaFold 3 | 2.58 | 2.63 | 0.92 | | 0.81 | 0.91 | 0.753 |
| 8TZX | Boltz-2 | 2.52 | 2.58 | 0.93 | | 0.95 | 0.97 | 0.739 |
| 8U17 | AlphaFold 3 | 5.92 | 6.13 | 0.78 | | 0.76 | 0.73 | 0.281 |
| 8U17 | Boltz-2 | 5.98 | 6.04 | 0.91 | | 0.91 | 0.91 | 0.255 |
| 9DJT | AlphaFold 3 | 2.91 | 2.95 | 0.92 | | 0.81 | 0.92 | 0.837 |
| 9DJT | Boltz-2 | 2.90 | 2.90 | 0.93 | | 0.94 | 0.96 | 0.855 |
| 9DJX | AlphaFold 3 | 2.98 | 3.17 | 0.92 | | 0.81 | 0.91 | 0.737 |
| 9DJX | Boltz-2 | 3.03 | 3.08 | 0.93 | | 0.94 | 0.96 | 0.74 |
| 9O91 | AlphaFold 3 | 0.68 | 0.68 | 0.93 | | 0.93 | 0.92 | 0.912 |
| 9O91 | Boltz-2 | 0.71 | 0.81 | 0.98 | | 0.98 | 0.97 | 0.909 |
| 9DOM | AlphaFold 3 | 0.72 | 0.95 | 0.86 | | 0.89 | 0.86 | 0.89 |
| 9DOM | Boltz-2 | 0.73 | 0.93 | 0.97 | | 0.96 | 0.95 | 0.82 |
| 8RQC | AlphaFold 3 | 0.91 | 0.97 | 0.91 | | 0.91 | 0.88 | 0.845 |
| 8RQC | Boltz-2 | 1.04 | 1.09 | 0.95 | | 0.97 | 0.97 | 0.823 |
